# The association between the genetic polymorphisms of LMP2/LMP7 and the outcomes of HCV infection among drug users^☆^

**DOI:** 10.1016/S1674-8301(10)60050-4

**Published:** 2010-09

**Authors:** Qian Cui, Yongxiang Zhang, Jing Su, Chao Shi, Na Lei, Keqin Ding, Jun Li, Rongbin Yu, Lu Wang, Ning Wang

**Affiliations:** a Department of Epidemiology and Biostatistics, School of Public Health, Nanjing Medical University, Nanjing 210029, Jiangsu Province, China; b Department of Infectious Diseases, the First Affiliated Hospital of Nanjing Medical University, Nanjing 210029, Jiangsu Province, China; c Department of Epidemiology, National Center for AIDS/STD Control and Provention, Chinese Center for Disease Control and Prevention, Beijing 100050, China

**Keywords:** hepatitis C virus, *LMP* gene, polymorphism, infection, outcome

## Abstract

**Objective:**

To investigate a possible association of *LMP2/LMP7* genes with chronic hepatitis C virus (HCV) infection, and to assess whether *LMP2/LMP7* genes could influence the outcomes of HCV infection among drug users.

**Methods:**

Genomic DNAs of 362 anti-HCV sero-positive drug users and 225 control drug users were extracted from the peripheral blood leukocytes. The sero-positive patients were divided into those who had persistent infection and those who had spontaneously cleared the infection. Polymorphisms of *LMP* genes were determined by PCR combined with restriction fragment length polymorphism (RFLP).

**Results:**

The distribution of *LMP2* genotypes among the control, persistent infection and spontaneous clearance groups were not different. However, the *LMP7* codon 145 Gln/Lys, Lys/Lys, and Gln/Lys+Lys/Lys genotypes were found significantly more frequent in the persistent infection group than in control group (OR=1.75, 95%CI=1.06∼2.90; OR=3.16, 95%CI=1.23-8.12; OR = 1.94, 95%CI=1.21-3.12, respectively). Similarly, the frequencies of the codon 145 Gln/Lys, Lys/Lys, and Gln/Lys+Lys/Lys genotypes were found significantly more frequent in the persistent infection group than in the spontaneous clearance group (OR=1.64, 95%CI=1.04-2.57; OR=2.40, 95%CI=1.09-5.28; OR=1.76, 95%CI=1.15-2.69, respectively). Stratified analysis indicated that combined genotype Gln/Lys + Lys/Lys of the *LMP7* gene was related to an increasing susceptibility to HCV infection (OR=1.91, 95%CI=1.02-3.55; OR=2.19, 95%CI=1.24-3.89; OR=1.91, 95%CI=1.05-3.48, OR=2.86, 95%CI=1.41-5.78, respectively) and the risk of persistent HCV infection (OR=1.94, 95%CI=1.12-3.34; OR=2.02, 95%CI=1.21-3.38; OR=1.78, 95%CI=1.12-2.85, OR=2.23, 95%CI=1.09-4.58, respectively) among > 30-year-old, males, the injection drug user (IDU) subjects and/or the shorter duration drug users (≤5 y).

**Conclusion:**

These results suggest that polymorphism of the *LMP7* gene may have an influence on the outcomes of HCV infection, and is one of the factors accounting for the genetic susceptibility to HCV infection among drug users.

## INTRODUCTION

Hepatitis C virus (HCV) infection is a major public health problem with a prevalence of 3.2% in the general population of China[1]. Moreover, the HCV prevalence rates were reported to be even higher among drug users, ranging from 18.30% to 66.97%[2]. Approximately 15%-25% of infected patients successfully eliminate the virus, whereas the remaining 70%-80% patients develop into chronic liver disease, including cirrhosis and hepatocellular carcinoma[3]. The variable outcome of HCV infection is influenced by many factors during the interactions between the virus and the host. Among these factors, variations in the genes involved in the immune response have already been linked to outcomes of HCV infection[4], presumably owing to alteration in the strength and quality of the immune response[5].

Major histocompatibility complex (MHC) class-I is a crucial molecule that initiates or regulates the immune response by presenting foreign- or self-antigens to T lymphocytes. Cytotoxic T lymphocyte-mediated immune responses are thought to play essential roles in the pathogenesis of liver injury by HCV and the reduction of viral load[6]. *LMP2/LMP7* genes which are located in the human MHC class-II DNA-binding protein 1 loci have been shown to be important in the MHC class-I antigen presentation pathway[7]. Products of *LMP2* and *LMP7* are distinct subunits of a multifunctional proteasome and play a pivotal role in selective degradation of endogenous proteins into peptides suited for binding to human MHC class-I molecule[8]. Previous studies have described limited polymorphisms in the coding regions of human *LMP2/LMP7* genes. These polymorphisms were found to be related to a number of immune diseases, including spondyloarthritis, juvenile rheumatoid arthritis (JRA), and malignant diseases[9]–[11]. Other reports have revealed that *LMP2/LMP7* genes are strongly correlated with the hepatitis B infection[12],[13]. Few studies, however, have examined the relationship between *LMP2/LMP7* genes and HCV infection. Therefore, we hypothesize that polymorphisms of *LMP2/LMP7* genes may alter the activities of the proteasome and influence the clinical course of hepatitis C. The aim of the present study was to investigate a possible association of the *LMP2/LMP7* genes with outcomes of HCV infection and determine whether these genes contribute to the susceptibility to HCV infection as a genetic risk factor among drug users.

## MATERIALS AND METHODS

### Study subjects

A total of 362 patients at the Nanjing Rehabilitation Center between May and Dec 2006 were recruited as the group of cases in this study. All patients were anti-HCV sero-positive, and then categorized into two subgroups for analysis: Group 1 consisted of 173 persistent HCV patients (123 males, 50 females; mean age 32.63±6.18 y), who were anti-HCV and HCV-RNA sero-positive with persistently normal or elevated ALT levels for at least three biochemical tests within six consecutive months during follow-up. Group 2 was composed of 189 spontaneous viral clearance patients (126 males, 63 females; mean age: 32.43±6.15 y), who were anti-HCV sero-positive and HCV-RNA sero-negative with persistently normal ALT levels (< 40 U/L) for all three tests. Anti-HCV and HCV RNA were conducted at least six months apart. The exclusion criteria of subjects included HBV or anti-HIV sero-positive, other causes of chronic liver disease, such as alcoholic liver diseases, or previous interferon and/or ribavirin therapy.

A total of 225 controls without HCV infection recruited from the same rehabilitation center were also enrolled as the control group (157 males, 68 females; mean age 31.81±8.09 y). Each participant was scheduled for an interview, and a structured questionnaire was administered by a trained interviewer to collect information on demographic data and environmental exposure history, including the method and duration of drug use, smoking and drinking. After the interview, an approximately 5 mL venous blood sample was collected from each participant.

### Viral testing

Anti-HCV was tested using a third generation enzyme-linked immunosobent assay (Intec Products Inc, China). Serum HCV-RNA was detected by reverse-transcription PCR (RT-PCR).

### SNP selection

*LMP2/LMP7* SNP candidates were selected by searching NCBI dbSNP database and related literatures. The criteria for selecting candidate SNPs include 1) SNPs with a minor allele frequency of more than 5% in the Chinese population, 2) SNPs located at the 5′-flanking region, 5′-untranslated region or exons with amino acid change (which were potentially functional polymorphisms of the *LMP2/LMP7* genes), and/or 3) reported SNPs associated with other diseases in Asians[11]–[13]. A total of two SNPs (rs17587 and rs2071543) met the above criteria.

### Genotyping

Genomic DNA from each subject was extracted from peripheral blood leukocytes by sodium dodecyl sulphate lysis and proteinase K digestion followed by a standard phenol-chloroform method according to a standard protocol[14]. The polymorphisms of *LMP2/LMP7* genes were detected by the PCR combined with restriction fragment length polymorphism (RFLP) analysis as previously described[15]. PCR was carried out in a 20-µL volume with 10×buffer [500 mmol/L KCl, 100 mmol/L Tris-HCl (pH 8.8), 25 mmol/L MgCl_2_], 1 pmol/L of each specific oligonucleotide primers, 100 µmol/L dNTPs, 50 ng of genomic DNA, and 1U *Taq* DNA polymerase. PCR was performed using a MJ-PTC-200 Thermal Cycler under specific PCR conditions. The reaction started at 95°C for 3 min. Thirty cycles of thermal cycling were completed following the conditions as denaturation at 95°C for 40 s, annealing at 57 and 55°C for 45 s, respectively (shown in ***Table 1***), extension at 72°C for 50 s, and a final extension was carried out at 72°C for 10 min. The amplified PCR products were digested using a specific restriction end nuclease (New England Biolabs, USA). After digestion, under different conditions recommended by the manufacturer, the fragments were electrophoresed on a 2%–3% agarose gel and visualized by ethidium bromide staining. The sizes of fragments were estimated by comparison with previously known size markers (Tiangen Biotech, China).

**Table 1:** Polymorphisms selection and primer sets used for the amplification of *LMP2/LMP7*

Gene	Position and substitution of the amino acid	PCR primers(forward/reverse)	Annealing temperature	Enzyme and fragment (bp)	SNP ID
*LMP2*	60(GCA→ACA) Arg→His	5′-GTGAACCGAGTGTTTGACAAGC-3′ 5′-GCCAGCAAGAGCCGAAAC AAG-3′	57°C	*Hin*6I(39 + 213)	rsl7587
*LMP7*	145(CAG→AAG) Gln→Lys	5′-TCATGGCGCTACTAGATGTATG-3′ 5′-AACTCTTTGTCCT AACTTGC AC-3′	55°C	*Bsm*I(146*+* 205)	rs2071543

Genotyping was performed without knowing the subjects' group status. Two research assistants read the gel pictures independently and repeated the assays if they did not reach a consensus on the tested genotype results. In addition, 10% of the samples were randomly selected to perform repeated assays, and the results were 100% concordant.

### Statistical analysis

The distribution of gender, injection drug user (IDU) and genotype frequencies between cases and controls were analyzed by using the χ^2^ test, and the age as well as duration of drug use by the Mann-Whitney test. Among controls, genotype frequencies for each SNP were tested for Hardy-Weinberg equilibrium. The association between genotypes and chronic HCV infection was estimated by computing the odds ratios (ORs) and 95% confidence intervals (CIs) from unconditional logistic regression analysis with adjustment for age, gender, IDU, and duration of drug use. A two-tailed *P*-value less than 0.05 was considered statistically significant. All the statistical analyses were performed using the SPSS software (Version 13.0, SPSS Inc, USA).

## RESULTS

### Basic characteristics

The basic characteristics of 225 controls, 189 patients with spontaneous clearance, and 173 patients with persistent infection are summarized in ***Table 2***. There were no significant differences in the distribution of age, gender, or *LMP2* allele frequencies among the three groups. However, statistical differences existed in terms of IDU, duration of drug use and *LMP7* allele frequencies among the three groups. The genotype distribution for *LMP2/LMP7* gene SNPs did not show any deviation from the Hardy-Weinberg equilibrium (all *P* > 0.05 in controls).

**Table 2:** Baseline characteristics of individuals included in the present study

Variables	Controls (*n =* 225)	Spontaneous clearance (*n* = 189)	Persistent infection (*n* = 173)	*P*
Age(mean±SD)	31.81±8.09	32.43±6.15	32.63±6.18	0.457^a^
Duration of drug use(mean±SD)	3.72±4.14	6.43±3.79	7.22±4.31	0.000 ^a^
Gender				
male	157(69.8)	126(66.7)	123(71.1)	
female	68(30.2)	63(33.3)	50(28.9)	0.639 ^b^
IDU				
yes	73(32.4)	161(85.2)	137(79.2)	
no	152(67.6)	28(14.8)	36(20.8)	0.000 ^b^
*LMP2*				
Arg	357(79.3)	295(78.5)	276(79.8)	
His	93(20.7)	81(21.5)	70(20.2)	0.906 ^b^
*LMP7*				
Gin	371(82.4)	233(67.3)	289(76.5)	
Lys	79(17.6)	113(32.7)	89(23.5)	o.ooo ^b^

a: one-way ANOVA; b: χ^2^ test.

[*n* (%)]

### Associations between genetic polymorphisms of *LMP2/LMP7* and susceptibility to HCV infection

Logistic regression analysis revealed that variant genotypes of the *LMP7* gene were observed to have statistically significant differences between the control group and the persistent HCV infection group. The variant genotypes Gln/Lys (adjusted OR = 1.75, 95%CI = 1.06-2.90), Lys/Lys (adjusted OR = 3.16, 95%CI = 1.23-8.12), and combined genotype Gln/Lys + Lys/Lys (adjusted OR = 1.94, 95%CI = 1.21-3.12) of *LMP7* were associated with significantly increased susceptibility to HCV compared with the Gln/Gln genotype. However, comparisons showed no significant correlation of *LMP2* gene genotypes with HCV infection (***Table 3***).

**Table 3:** Distribution of *LMP2/LMP7* genotypes in each study group

Genotypes	Controls [*n* (%)]	Spontaneous clearance [*n* (%)]	Persistent infection [*n* (%)]	Controls/Persistent infection			Controls/Spontaneous clearance		Persistent infection/Spontaneous clearance	
				P*	OR(95%CI)*		P*	OR(95%CI)*	P*	OR(95%CI)*
*LMP2*										
Arg/Arg	140(62.2)	113(59.8)	112(64.7)	—	l.00(reference)		—	l.00(reference)	—	l.00(reference)
Arg/His	77(34.2)	69(36.5)	52(30.1)	0.362	0.79(0.48-1.31)		0.947	1.02(0.62-1.66)	0.181	0.73(0.47-1.16)
His/His	8(3.6)	7 (3.7)	9(5.2)	0.497	1.53(0.45-5.19)		0.991	0.99(0.27-3.71)	0.995	0.99(0.35-2.85)
Arg/His+His/His	85(37.8)	76(40.2)	61(35.3)	0.505	0.85(0.52-1.38)		0.952	1.02(0.63-1.63)	0.217	0.76(0.49-1.18)
*LMP7*										
Gln/Gln	155(68.9)	112(59.3)	80(46.2)	—	l.00(reference)		—	l.00(reference)	—	l.00(reference)
Gln/Lys	61(27.1)	65(34.4)	73(42.2)	0.029	1.75(1.06-2.90)		0.810	1.07(0.64-1.77)	0.032	1.64(1.04-2.57)
Lys/Lys	9(4.0)	12(6.3)	20(11.6)	0.017	3.16(1.23-8.12)		0.343	1.68(0.57-4.97)	0.030	2.40(1.09-5.28)
Gln/Lys+Lys/Lys	70(31.1)	77(40.7)	93(53.8)	0.006	1.94(1.21-3.12)		0.604	1.14 (0.70-1.85)	0.010	1.76(1.15-2.69)

* Logistic regression model, adjusted by gender, age, injection drug user and duration of drug use.

### Associations between genetic polymorphisms of *LMP2/LMP7* and outcomes of HCV infection

There were also significant differences in the distribution of variant *LMP7* genotypes between the spontaneous clearance group and persistent HCV infection group. Compared with the Gln/Gln genotype, the risk of persistent infection for subjects with Gln/Lys (adjusted OR = 1.64, 95%CI = 1.04-2.57), Lys/Lys (adjusted OR = 2.40, 95%CI = 1.09-5.28), and combined genotype Gln/Lys + Lys/Lys (adjusted OR = 1.76, 95%CI = 1.15-2.69) were all significantly increased. No statistically significant differences were found for the genotypes of *LMP2* between the two HCV infected groups (***Table 3***).

### Stratified analysis

The stratified analysis showed the protective effect of the *LMP2* Arg/His + His/His genotype remained significant among subjects with shorter duration of drug use (≤5 y). In addition, we did not find any significant effect in the other strata (***Table 4***). However, the data showed that compared with the Gln/Gln genotype, the increased risks of HCV infection associated with *LMP7* gene combined genotype Gln/Lys+Lys/Lys (OR=1.91, 95%CI=1.02-3.55; OR=2.19, 95%CI=1.24-3.89; OR=1.91, 95%CI=1.05-3.48; OR=2.86, 95%CI=1.41-5.78, respectively) were more pronounced among the older subjects (> 30-year-old), the males, and the IDU and the shorter duration of drug use (≤5 y). The results also indicated that combined genotype Gln/Lys+Lys/Lys was associated with increased risks of persistent HCV infection: the older subjects (OR=1.94, 95%CI=1.12-3.34), the males (OR=2.02, 95%CI=1.21-3.38), the IDU (OR=1.78, 95%CI=1.12-2.85), and the shorter duration of drug use (OR=2.23, 95%CI=1.09-4.58, ***Table 5***).

**Table 4:** Stratified analysis of *LMP2* gene codon 60 genotypes with chronic HCV infection

Stratified characteristics	Persistent infection [*n* (%)]		Spontaneous clearance [*n* (%)]		Controls [*n* (%)]		Controls/Persistent infection OR(95%CI)*		Controls/Spontaneous clearance OR(95%CI)*		Persistent infection/Spontaneous clearance OR(95%CI)*	
	Arg/Arg	Arg/His +His/His	Arg/Arg	Arg/His +His/His	Arg/Arg	Arg/His +His/His	Arg/Arg	Arg/His +His/His	Arg/Arg	Arg/His +His/His	Arg/Arg	Arg/His +His/His
Age (y)												
≤30	38(67.9)	18(32.1)	52(63.4)	30(36.6)	63(60.0)	42(40.0)	1.00	0.83(0.38-1.82)	1.00	1.15(0.56-2.36)	1.00	0.81(0.39-1.68)
>30	74(63.2)	43(36.8)	61(57.0)	46(43.0)	77(64.2)	43(35.8)	1.00	0.83(0.45-1.54)	1.00	0.97(0.51-1.85)	1.00	0.73(0.42-1.26)
Gender												
male	79(64.2)	44(35.8)	76(60.3)	50(39.7)	95(60.5)	62(39.5)	1.00	0.87(0.49-1.55)	1.00	0.90(0.50-1.60)	1.00	0.80(0.47-1.35)
female	33(66.0)	17(34.0)	37(58.7)	26(41.3)	45(66.2)	23(33.8)	1.00	0.65(0.26-1.62)	1.00	1.25(0.53-2.97)	1.00	0.67(0.30-1.49)
IDU												
yes	91(66.4)	46(33.6)	95(59.0)	66(41.0)	42(57.5)	31(42.5)	1.00	0.68(0.37-1.26)	1.00	1.04(0.59-1.84)	1.00	0.67(0.41-1.09)
no	21(58.3)	15(41.7)	18(54.3)	10(35.7)	98(64.5)	54(35.5)	1.00	1.09(0.50-2.37)	1.00	0.91(0.38-2.16)	1.00	1.24(0.43-3.59)
Duration of drug use (y)												
≤5	48(81.4)	11(18.6)	46(59.7)	31(40.3)	95(61.3)	60(38.7)	1.00	0.25(0.11-0.59)	1.00	0.74(0.38-1.46)	1.00	0.35(0.16-0.78)
5∼10	32(54.2)	27(45.8)	53(66.2)	27(33.8)	26(59.1)	18(40.9)	1.00	1.52(0.63-3.66)	1.00	0.84(0.35-2.00)	1.00	1.73(0.82-3.64)
≥10	32(58.2)	23(41.8)	14(43.8)	18(56.2)	19(73.1)	7(26.9)	1.00	2.26(0.76-6.76)	1.00	2.76(0.81-9.39)	1.00	0.54(0.22-1.34)

* Logistic regression model, adjusted by gender, age, injection drug user, and duration of drug use.

**Table 5:** Stratified analysis of *LMP7* gene codon 145 genotypes with chronic HCV infection

Stratified characteristics		Persistent infection [*n* (%)]			Spontaneous Clearance [*n* (%)]		Controls [*n* (%)]		Controls/Persistent infection OR(95%CI)*		Controls/Spontaneous clearance OR(95%CI)*		Persistent infection/Spontaneous clearance OR(95%CI)*	
		Gln/Gln	Gln/Lys +Lys/Lys		Gln/Gln	Gln/Lys +Lys/Lys	Gln/Gln	Gln/Lys +Lys/Lys	Gln/Gln	Gln/Lys +Lys/Lys	Gln/Gln	Gln/Lys +Lys/Lys	Gln/Gln	Gln/Lys +Lys/Lys
Age (y)														
≤30		26(46.4)	30(53.6)		45(54.9)	37(45.1)	71(67.6)	34(32.4)	1.00	2.13(0.99-4.56)	1.00	1.20(0.59-2.44)	1.00	1.47(0.74-2.95)
>30		54(46.2)	63(53.8)		67(62.6)	40(37.4)	84(70.0)	36(30.0)	1.00	1.91(1.02-3.55)	1.00	1.11(0.57-2.16)	1.00	1.94(1.12-3.34)
Gender														
male		54(43.9)		69(56.1)	76(60.3)	50(39.7)	109(69.4)	48(30.6)	1.00	2.19(1.24-3.89)	1.00	1.03(0.57-1.86)	1.00	2.02(1.21-3.38)
female		26(52.0)		24(48.0)	36(57.1)	27(42.9)	46(67.6)	22(32.4)	1.00	1.47(0.61-3.56)	1.00	1.25(0.52-2.99)	1.00	1.30(0.60-2.82)
IDU														
yes		60(43.8)		77(56.2)	93(57.8)	68(42.2)	45(61.6)	28(38.4)	1.00	1.91(1.05-3.48)	1.00	1.15(0.64-2.04)	1.00	1.78(1.12-2.85)
no		20(55.6)		16(44.4)	19(67.9)	9(32.1)	110(72.4)	42(27.6)	1.00	1.97(0.90-4.33)	1.00	1.07(0.43-2.65)	1.00	1.63(0.57-4.62)
Duration of drug use (y)														
≤5		28(47.5)		31(52.5)	50(64.9)	27(35.1)	112(72.3)	43(27.7)	1.00	2.86(1.41-5.78)	1.00	1.22(0.61-2.47)	1.00	2.23(1.09-4.58)
5∼10		26(44.1)		33(55.9)	41(51.2)	39(48.8)	29(65.9)	15(34.1)	1.00	1.86(0.77-4.45)	1.00	1.50(0.63-3.56)	1.00	1.51(0.74-3.10)
≥10		26(47.3)		29(52.7)	21(65.6)	11(34.4)	14(53.8)	12(46.2)	1.00	1.18(0.43-3.23)	1.00	0.48(0.14-1.65)	1.00	2.40(0.93-6.21)

* Logistic regression model, adjusted by gender, age, injection drug user and duration of drug use.

## DISCUSSION

The clinical course of HCV infection varies substantially among individuals. An increased and broadly multispecific T-cell response is thought to be critical to a favorable outcome[16]. The antigen recognition by cytotoxic CD_8_^+^ T cells is dependent upon a number of crucial steps in antigen processing, including LMP2 and LMP7 that can alter the pool of peptides available for class I antigen presentation through enhanced substrate cleavage after basic and hydrophobic amino acid residues compared to the constitutive proteasome catalytic subunits[17]. This process has the potential to shape the CD_8_^+^ T cell response to viral antigens both by increasing the diversity of produced peptides and by favoring the production of peptides with carboxylterminal amino acid residues that more tightly bind MHC-I molecules. Therefore, genetic variation of *LMP2/LMP7* might play an important role in the immunological reaction to HCV infection.

Recently, several associations have been reported between *LMP2/LMP7* genes and some diseases[9]–[11]. However, to our knowledge, few studies have reported on the association of *LMP2/LMP7* genes with chronic HCV infection. One report from Japan revealed that *LMP7*-145 SNP is one of the important host factors which independently influences the response to IFN in patients with chronic hepatitis C[18]. Our present study is the first to extensively explore the association between genetic polymorphisms of *LMP2/LMP7* and the outcomes of HCV infection among drug users.

In this study, we observed statistically significant differences between persistent HCV infection patients and control subjects in the distributions of *LMP7*-145 genotype. Compared with *LMP7* codon145 Gln/Gln genotype, we found that Gln/Lys, Lys/Lys, and combined genotype Gln/Lys+Lys/Lys could increase the risk of HCV infection. Our results are in agreement with previous reports that demonstrated that codon145 (Gln>Lys) polymorphism of *LMP7* gene was one of the important susceptibility factors of HBV infection[12]–[13]. Similarly, our study also revealed that SNP at codon145 of the *LMP7* gene might relate to the outcomes of HCV infection, and compared with carrying Gln/Gln homozygote, polymorphism of codon145 Gln>Lys could increase the risk of persistent HCV infection in patients. These findings suggested that LMP7-Lys may be a potential genetic marker of HCV infection. In addition, stratified analysis showed the higher risk of susceptibility to HCV infection or persistence associated with *LMP7* codon145 combined genotype Gln/Lys+Lys/Lys was more pronounced among the older subjects, males, the IDU, and the shorter duration of drug use. The results suggested that the effects of *LMP7* were not equivalent in all individuals. For subjects with certain characteristics, *LMP7* may have a more dominant effect on the clinical course of an HCV infection. The increased risk could be due to the changed activity of *LMP7*, or the additional contribution by other factors rather than *LMP7*. Nevertheless, the true mechanism leading to this changed relationship between *LMP7* and HCV is not clear yet.

Our study also showed the polymorphism at codon 60 of *LMP2* gene seemed to be irrelevant to the outcome of an HCV infection. This observation was in good agreement with previous studies, which demonstrated that there was no association between *LMP2* codon 60 polymorphism and a series of diseases[11],[19]. But interestingly, in the stratified analysis, the protective effect of the *LMP2* Arg/His+His/His genotypes seemed significant in individuals with a shorter duration of drug use (≤5 y). The reason may be the interaction between the shorter duration of drug use and *LMP2* gene and/or the effect of other virulence factors. However, this relationship needs to be confirmed.

The present study still has several limitations: First, the selection bias between cases and controls was inevitable, and the subjects may not be representative of the general population. Second, several other factors such as age at acquisition of HCV infection, gender, ethnicity, and co-infection with HBV or HIV have also been involved in the development of HCV infection[20],[21]. It is difficult to exclude or estimate the influence of these factors in this study, except where they were exclusion criteria in subject selection. Besides, because of the small sample size in stratified subjects, these findings are preliminary, and a large-scale study needs to be performed to assure the potential of *LMP2/LMP7* gene for prevention and management of HCV infection.

In conclusion, our findings implicate genetic variants of *LMP7* playing an important role in determining the susceptibility to HCV and having an effect on the variable outcomes of HCV infection among drug users.
